# Metabolic heterogeneity in cancer

**DOI:** 10.1186/2049-3002-2-S1-O1

**Published:** 2014-05-28

**Authors:** Ralph J DeBerardinis

**Affiliations:** 1 Children’s Medical Center Research Institute, UT Southwestern Medical Center, USA

## 

Metabolic reprogramming has long been viewed as an important component of malignant transformation. Recent years have defined a large number of mechanisms by which oncogene-directed perturbations of signal transduction regulate intermediary metabolism, and conversely, by which metabolites themselves influence gene expression and other dimensions of systems biology. However, there is still little agreement as to the breadth of metabolic programs that can support cancer cell growth, and more importantly, about which of the myriad metabolic activities observed in culture models of cancer cell proliferation are relevant to the survival and growth of tumors *in vivo*. I will discuss approaches to address these two challenges. First, I will discuss ongoing efforts to link functional metabolic pathway choices with oncogenotypes using systematic analysis of large panels of cancer cell lines. Second, I will describe efforts to probe metabolism in living tumors from humans and mice, using a combination of stable isotope tracing and other methods.

